# Supplementary material for: Estimation of effective number of breeders and effective population size in an abundant and heavily exploited marine teleost
Source: Evol Appl. 2024 Jul 22;17(7):e13758. doi: 10.1111/eva.13758 (PMC11261160; doi:10.1111/eva.13758)
Supplement: Supplementary file 1 — Data S1. [file EVA-17-e13758-s001.docx]

**Table S1** Numbers of SNPs retained after each bioinformatic filtering step.

| Step | SNP count |
| --- | --- |
| Raw SNP catalogue | 7,338,377 |
| 80% of individuals, biallelic, >0.03 minor allele frequency | 45,715 |
| Remove indels | 41,513 |
| Read quality (ratio quality/coverage depth >0.2) | 40,935 |
| Mapping quality (>30) | 37,041 |
| High coverage loci (≤mean depth + (2*standard deviation)) | 35,992 |
| Hardy-Weinberg equilibrium in >67% of locations | 31,275 |
| Call error rate (0.95) | 27,794 |
| Linkage disequilibrium (500) | 6,839 |
| Putatively neutral loci | 6,818 |

**Table S2** Pairwise *F*_ST_ values (i.e., genetic differentiation) between all snapper samples, with significant comparisons denoted with an asterisk. South-west samples are: CS, Cockburn Sound; CS14, Cockburn Sound 2014 sample; BUS, Busselton; ALB, Albany; CSYOY, south-west YOY. South-east samples are: KSE, Kingston SE; PLD, Portland; PPB, Port Phillip Bay; PPB11, Port Phillip Bay 2011 samples; WPB, Western Port Bay; PPBYOY, south-east YOY.

|  | CS | CS14 | BUS | ALB | CSYOY | KSE | PLD | PPB | PPB14 | WPB | PPBYOY |
| --- | --- | --- | --- | --- | --- | --- | --- | --- | --- | --- | --- |
| CS | 0 |  |  |  |  |  |  |  |  |  |  |
| CS14 | -0.0007 | 0 |  |  |  |  |  |  |  |  |  |
| BUS | -0.0003 | -0.0004 | 0 |  |  |  |  |  |  |  |  |
| ALB | 0.0024^*^ | 0.0013 | 0.0012 | 0 |  |  |  |  |  |  |  |
| CSYOY | 0.0000 | -0.0004 | 0.0001 | 0.0021^*^ | 0 |  |  |  |  |  |  |
| KSE | 0.0250^*^ | 0.0259^*^ | 0.0249^*^ | 0.0232^*^ | 0.0266^*^ | 0 |  |  |  |  |  |
| PLD | 0.0255^*^ | 0.0250^*^ | 0.0243^*^ | 0.0232^*^ | 0.0264^*^ | 0.0008 | 0 |  |  |  |  |
| PPB | 0.0257^*^ | 0.0261^*^ | 0.0255^*^ | 0.0244^*^ | 0.0275^*^ | 0.0002 | 0.0010 | 0 |  |  |  |
| PPB14 | 0.0249^*^ | 0.0255^*^ | 0.0245^*^ | 0.0232^*^ | 0.0264^*^ | 0.0007 | 0.0007 | 0.0005 | 0 |  |  |
| WPB | 0.0237^*^ | 0.0235^*^ | 0.0229^*^ | 0.0224^*^ | 0.0253^*^ | 0.0006 | 0.0006 | 0.0004 | 0.0004 | 0 |  |
| PPBYOY | 0.0250^*^ | 0.0250^*^ | 0.0244^*^ | 0.0233^*^ | 0.0263^*^ | 0.0003 | 0.0005 | 0.0002 | 0.0004 | -0.0001 | 0 |

**
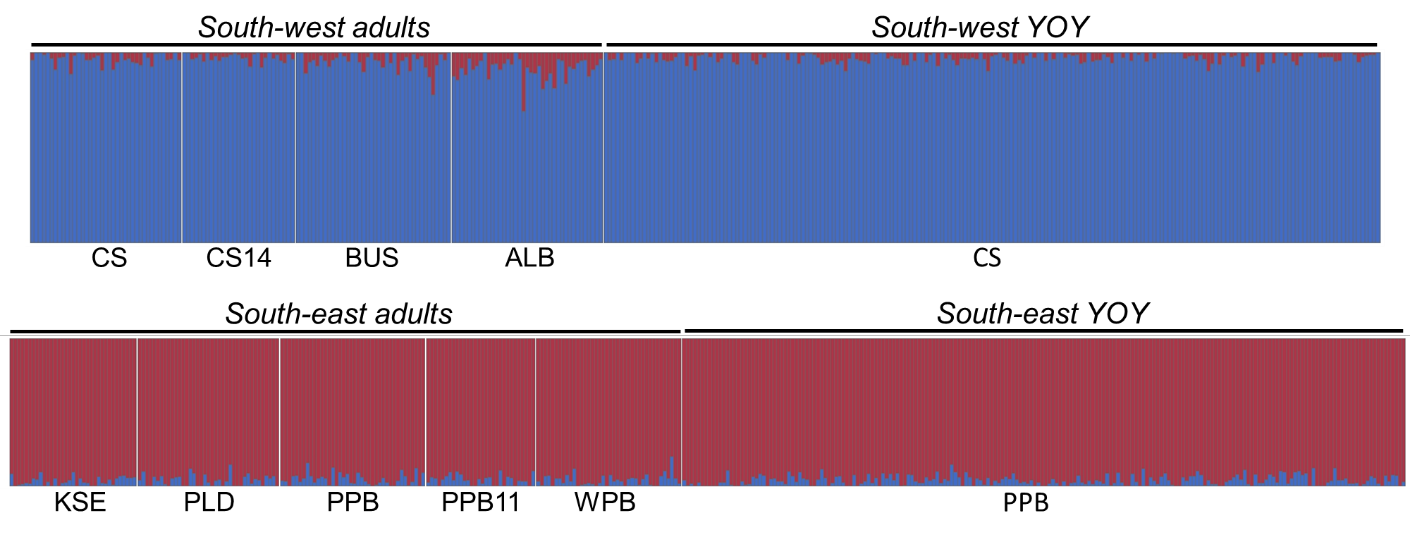
**

**Figure S1** ADMIXTURE results for *K* = 2 based on 6,818 neutral SNPs illustrating the genetic affinity between all young of the year (YOY) and adult snapper samples collected from the same geographical area. Each vertical bar represents an individual, and each individual’s colour makeup signifies its probability of membership to each of the two genetic clusters (represented by blue and red). CS, Cockburn Sound; BUS, Busselton; ALB, Albany; KSE, Kingston SE; PLD, Portland; PPB, Port Phillip Bay; WPB, Western Port Bay.


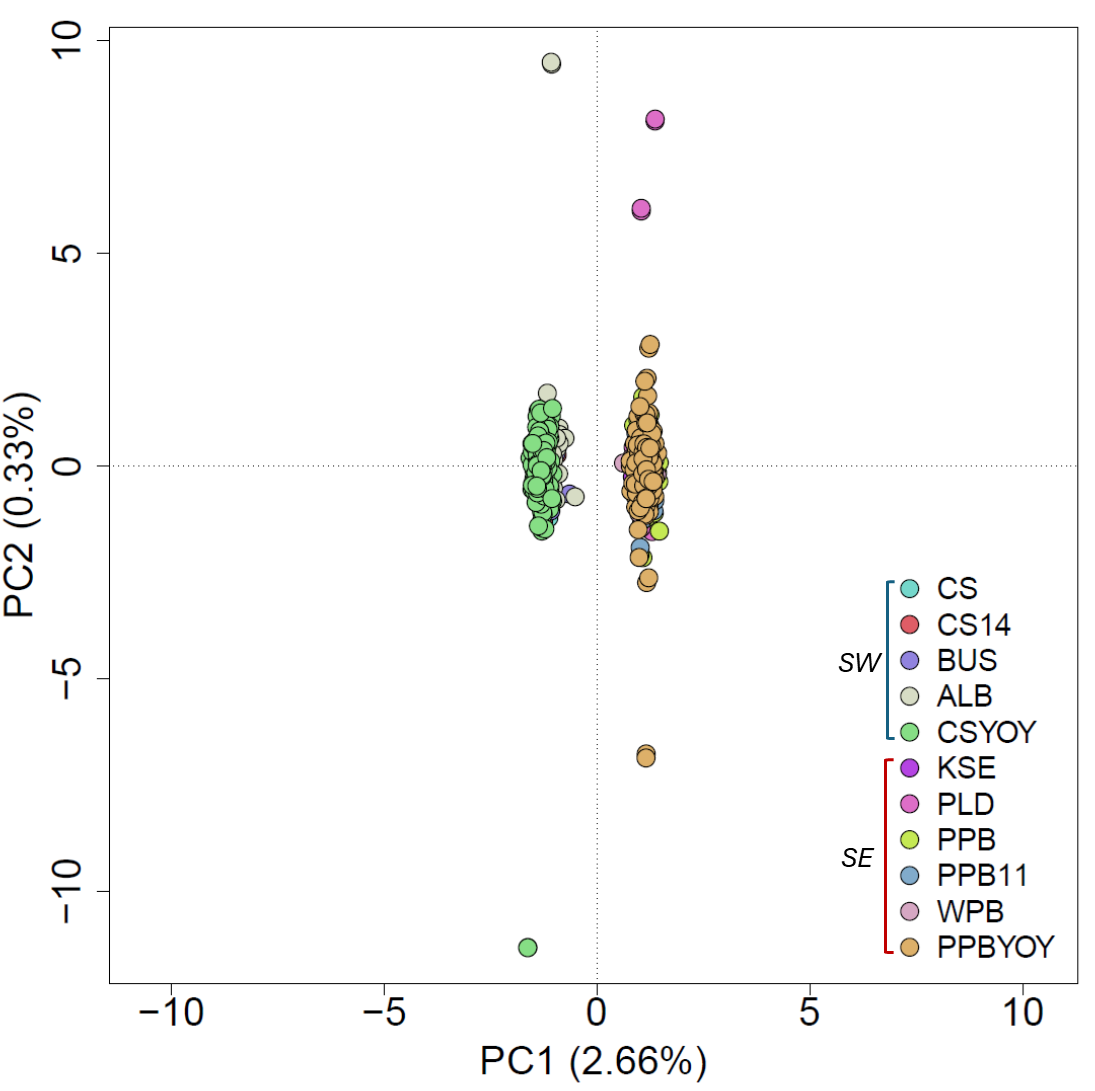


**Figure S2** Principal component analysis (PCA) of genetic differentiation, with principal component 1 (PC1: 2.66% of variance) against principal component 2 (PC2: 0.33% of variance). Each point represents an individual, which are colour coded by sample type. South-west (SW) samples are: CS, Cockburn Sound; CS14, Cockburn Sound 2014 sample; BUS, Busselton; ALB, Albany; CSYOY, south-west YOY. South-east (SE) samples are: KSE, Kingston SE; PLD, Portland; PPB, Port Phillip Bay; PPB11, Port Phillip Bay 2011 samples; WPB, Western Port Bay; PPBYOY, south-east YOY.
